# Supplementary material for: Sulphated TiO2 Reduced by Ammonia and Hydrogen as an Excellent Photocatalyst for Bacteria Inactivation
Source: Materials (Basel). 2023 Dec 22;17(1):66. doi: 10.3390/ma17010066 (PMC10779939; doi:10.3390/ma17010066)
Supplement: Supplementary file 1 [file materials-17-00066-s001.zip › materials-2769454-supplementary.pdf]

Supplementary material for:

Sulphated TiO_2 Reduced by Ammonia and Hydrogen as An Excellent Photocatalyst for Bacteria Inactivation

Piotr Rychtowski[1*], Oliwia Paszkiewicz[2], Agata Markowska-Szczupak[2], Grzegorz Leniec[3] and Beata Tryba[1]

[1] Department of Catalytic and Sorbent Materials Engineering, Faculty of Chemical Technology and Engineering, West Pomeranian University of Technology in Szczecin, Pułaskiego 10, 70-322 Szczecin, Poland.
[2] Department of Chemical and Process Engineering, West Pomeranian University of Technology, Piastów 42, 71-065 Szczecin, Poland.
[3] Department of Nanomaterials Physicochemistry, Faculty of Chemical Technology and Engineering, West Pomeranian University of Technology, Szczecin, Piastów Ave. 42, 71-065 Szczecin, Poland.
* Correspondence: piotr.rychtowski@zut.edu.pl

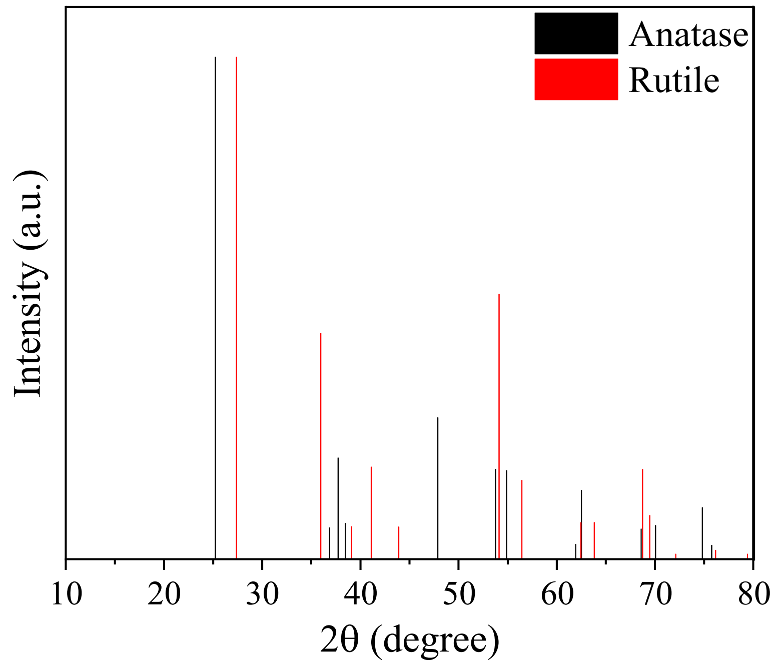

Figure S1. XRD reference patterns of anatase (black pattern) and rutile (red pattern)

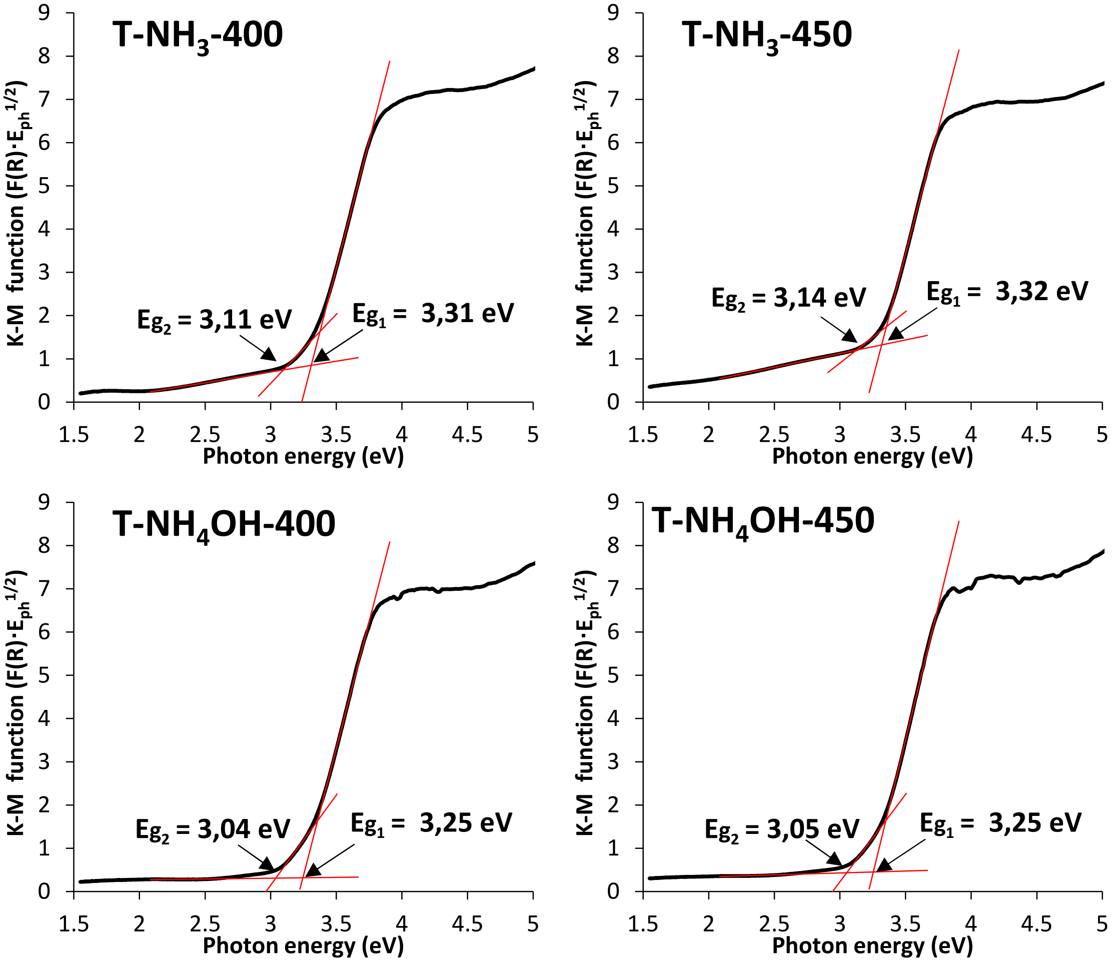

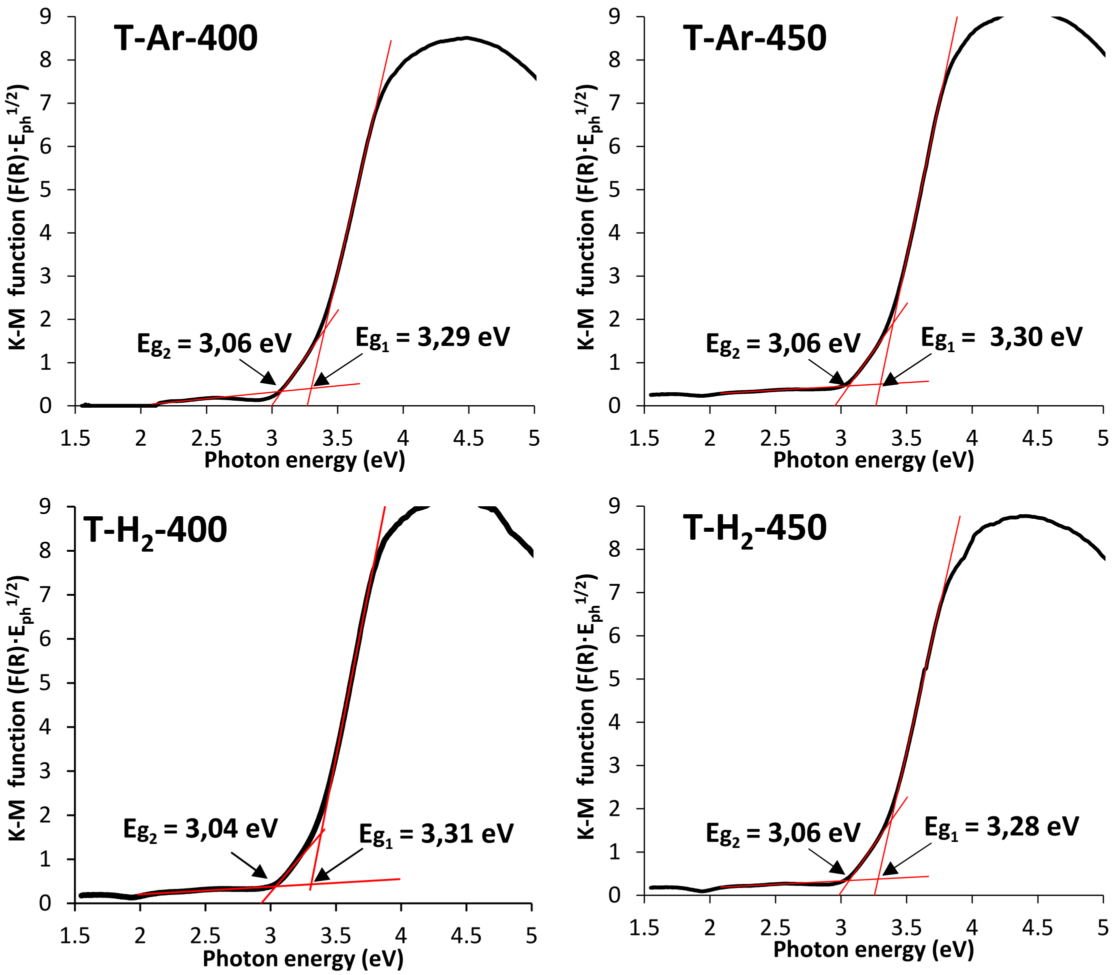

Figure S2. Kubelka-Munk graphs for band-gap values determination of TiO_2-based photocatalysts heat-treated in 400-450 °C
